# Thermal Desorption and Extraction Coupled With Gas Chromatography and Mass Spectrometry for the Quantification of Polystyrene Nanoplastic in Pak Choi

**DOI:** 10.1002/rcm.10046

**Published:** 2025-04-14

**Authors:** Eric Zytowski, Susanne Baldermann

**Affiliations:** ^1^ Leibniz Institute for Vegetable and Ornamental Crops (IGZ) Grossbeeren Germany; ^2^ Institute of Nutritional Science University Potsdam Nuthetal Germany; ^3^ Faculty for Life Sciences: Food, Nutrition and Health, Food Metabolome, Kulmbach University of Bayreuth Bayreuth Germany

**Keywords:** crops, micro‐ and nanoplastics, plastic accumulation, polystyrene, TED‐GC/MS

## Abstract

**Rationale:**

It has been demonstrated that microplastics and nanoplastics (MNPs) can be found in soil and that MNPs can be taken up by plants. In order to conduct a risk assessment for human consumption, it is necessary to have an estimate of the mass concentration of plastics in crops. A new thermal extraction and desorption coupled with gas chromatography and mass spectrometry (TED‐GC/MS) method has been developed for the analysis of polystyrene (PS) in pak choi.

**Methods:**

In this study, a thermogravimetric analyser (TGA) equipped with a thermal absorption unit (TAU), was coupled with a GC system equipped with a thermal desorption unit (TDU2), a (5%‐phenyl)‐methylpolysiloxane GC column and a GC/MSD single quadrupole mass spectrometer. The systems were connected via an MultiPurposeSampler (MPS). Samples were pyrolyzed in the TGA; the pyrolysis products were trapped on a PDMS polymer bar, desorbed in the TDU, separated and analysed on the GC/MS system.

**Results:**

The purpose of this study was to investigate the qualitative and quantitative detection of PS MNPs in pak choi. The determined limit of detection (LOD) was 0.09 μg, and the limit of quantification (LOQ) was 0.28 μg PS absolute. Plants treated with 100 nm of particles 19.0 ± 6.7 μg/g DM PS and in the plants treated with 500 nm of particles 64.1 ± 8.6 μg/g DM PS have been found.

**Conclusions:**

This study was the first to use a TED‐GC/MS method for the detection of PS nanoplastics of different sizes in pak choi and thus provides an important basis for the determination and risk assessment of PS in vegetables.

## 1 Introduction

Microplastics and nanoplastics (MNPs) can be found ubiquitous in the environment [1, 2, 3, 4]. In 2022, worldwide plastic production reached 400.3 million tonnes, with 58.7 million tonnes produced in Europe [5]. A study estimated that between the years 1950 and 2015, 4900 million tons of plastic waste were deposited in landfills or the environment [6]. The discovery of MNPs in agricultural soils raises the question of whether it is taken up by plants and, if so, in what quantities [2]. Due to a lack of quantitative methods, the development of new analytical techniques is essential for the analysis of MNPs in plant material and for the estimation of MNPs concentrations in vegetables intended for human consumption, as well as for the assessment of associated risks [7].

The most significant challenges in the analysis of microplastics (1–1000 μm) and nanoplastics (1–1000 nm) are the different particle sizes, the high chemical diversity of the plastics and the separation the from matrix [8]. Analysing MNPs in plants, optical methods like confocal laser scanning microscopy (CLSM) or scanning electron microscopy (SEM) are used for the localisation and particle size detection of MNPs in plants, while pyrolysis gas chromatography coupled with mass spectrometry (pyr‐GC/MS) is used for identification and quantification of MNPs [8, 9, 10, 11]. However, microscopic techniques, for example, provide no information about the chemical composition and properties of the polymer, and small and transparent particles are difficult to detect. Matrix effects are reported for Fourier transform infrared (FTIR), Raman or nuclear magnetic resonance (NMR) spectroscopy, which may limit their application and require sample preparation. The sensitivity of the existing mass‐based methods can be insufficient and enrichment methods need to be developed, which may also lead to changes of MNPs [7]. It is imperative to minimise MNPs contamination, while ensuring that the extraction process does not affect their degradation or change the surface and chemical properties of the particles. A study examined the impact of an acidic and an alkaline matrix digestion protocol, with the results indicating that the alkaline protocol exerts a more significant influence on polymer properties, such as degradation [12]. In accordance with the regulatory DIN EN ISO 24187:2024‐04 standard, the analytical methods for MNPs encompassed the use of Pyr‐GC/MS and thermal extraction and desorption gas chromatography coupled with mass spectrometry (TED‐GC/MS) [13].

The TED‐GC/MS is a thermoanalytical method for qualitative and quantitative analysis of MNPs in different matrices. In this model study, pak choi was used as leafy vegetable and polystyrene as common plasticto develop a method to quantitate MNPs in plants. Pak choi serves as a model vegetable, with all above‐ground parts suitable for consumption. Heavy metals and pesticides have been identified as potential contaminants in Brassica vegetables [14, 15]. The lack of data on MNPs as contaminants in vegetables such as pak choi has prompted the development of new analytical methods for quantitative analysis and risk assessment in this study and other studies [16, 17]. Notably, indoor cultivation carries a high risk of contamination with plastics, as the majority of equipment used is made of plastic [18].

**FIGURE 1:**
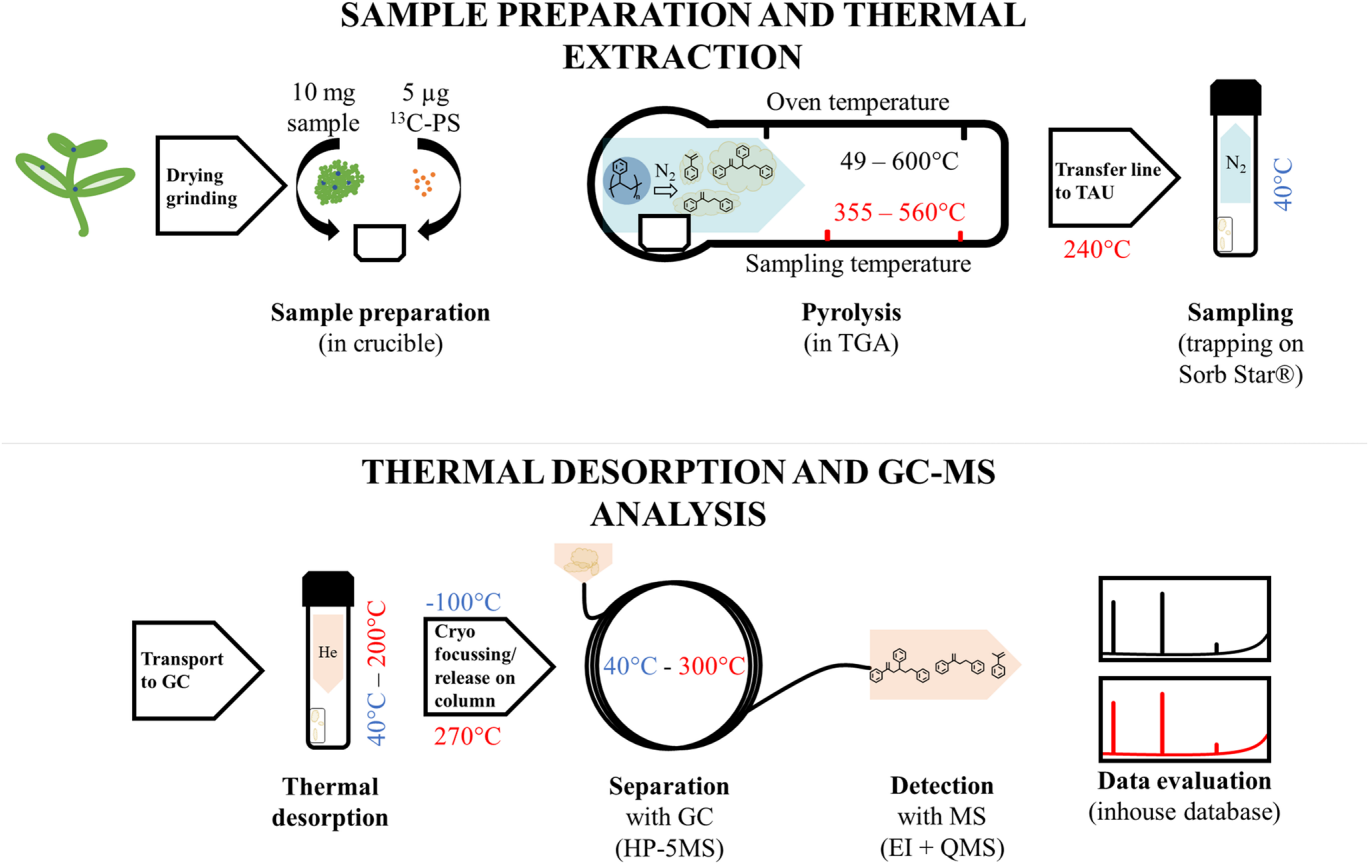
Workflow of TED‐GC/MS analysis of PS in the pak choi matrix.

The sample preparation for the new TED‐GC/MS method for the analysis of MNPs in vegetables is very simple and contains less steps to avoid contamination from labware, than the highly processed samples for Pyr‐GC/MS. The complete workflow of the new developed method is shown in Figure 1.

## 2 Material and Methods

### 2.1 Samples and Chemical Reagents

Polymer reference standards including polyethylene (PE), polyethylene terephthalate (PET), polypropylene (PP), polystyrene (PS), polyvinylchloride (PVC), styrene butadiene rubber (SBR) were purchased from BAM (Berlin, Germany); polytetrafluoroethylene (PTFE) was purchased from Sigma‐Aldrich (Taufkirchen, Germany); and polylactic acid (PLA) was purchased from Goodfellow GmbH (Hamburg, Germany). As internal standard (IS), an isotope labelled PS (^13^C_6_) was used from Sigma‐Aldrich (Taufkirchen, Germany). PS‐MNPs in aqueous solution with defined sizes (100 nm, 25 mg/mL; 500 nm, 50 mg/mL; 2 μm, 50 mg/mL; 5 μm, 50 mg/mL) were purchased from micromod (Rostock, Germany) and diluted with Millipore water to obtain 1 μg/μL working solutions. In order to ascertain the impact of particle size on the quantitative outcomes, 5 μg of PS‐MNPs (100 and 500 nm and 2 and 5 μm) and 3 μg of IS were subjected to analysis in triplicate.

### 2.2 Plant Material

Seeds of pak choi (*Brassica rapa subsp. chinensis cv*. Black Behi) were grown on a 0.5 *wt*.% PS‐soil‐mixture (Einheitserde type P, pH 5.8, Germany; PS‐MNPs: 100 and 500 nm) under controlled conditions in a controlled climate cabinet (temperature: 22°C, day/night: 16 h/8 h, light intensity: 100 μmol/(m^2^s)). To avoid plastic contamination, plants were cultivated in small glass beakers. Gardening supplies were replaced with plastic‐free materials, such as glass beakers for watering and fertilising and aluminium foil for harvesting. The plant samples were harvested 6 weeks after growth by cutting the plants above the soil to prevent soil contamination. The samples were then collected in aluminium foil and immediately frozen in liquid nitrogen. Plastic‐free plants (control) were grown in the same manner, without adding PS to the soil. The freeze‐dried plant material was pulverised with steel balls in metal vessels using a mixer mill MM400 from Retsch (Düsseldorf, Germany).

Plant samples ( *Solanum lycopersicum* , *Nicotiana tabacum* , *Salicornia europaea* and *Asparagus officinalis* ) were obtained for testing the interference with PS. These samples were obtained from experimental controls using comparable growing conditions from experiments conducted at the institute.

### 2.3 Coupling Thermal Extraction With Thermal Desorption and GC/MS Analysis

#### 2.3.1 Sample Preparation

The 150 μL of aluminium oxide crucibles from Mettler Toledo (Gießen, Germany) was baked at 1000°C for 6 h under an oxygen atmosphere to eliminate polymer residues. Approximately 10 mg of powdered samples was added after the baking process, along with 5 μg of IS (1 μg/μl ^13^C_6_‐PS dissolved in toluene). To create the calibration curves, 10 mg of powdered, plastic‐free pooled control samples was spiked with 1 μg/μL PS solution in toluene (respectively, 0.1, 0.2, 0.3, 0.5, 0.7, 1.0 and 1.5 μg), along with 5 μg of the IS solution. Quality controls (QC) were prepared as described above, with 0.5 μg of PS, 5 μg of IS and 10 mg of control samples added to the crucibles. The samples and QC were placed afterwards in the auto sampler of the TGA.

#### 2.3.2 Thermal Extraction and Desorption

The thermal extraction was carried out using a thermogravimetric analyser (TGA2) from Mettler Toledo (Zurich, Switzerland) under a nitrogen atmosphere. The heating rate was set to 10°C/min, and the temperature range was from 49°C to 600°C. Pyrolysis products were trapped between 355°C and 560°C TGA temperature, based on the TGA curves of the pure polymers (Figure S1) and to reduce the matrix. The analytes were transferred from the TGA using a heated coupling device (240°C) under a nitrogen flow of 80 mL/min and trapped in a thermal absorption unit (TAU) from GERSTEL (Mülheim, Germany) using Sorb Star® polymer bars (20‐mm length, 2‐mm diameter) purchased from ENVEA GmbH (Vohenstrauß, Germany) in glass tubes at 40°C. The glass tubes containing the Sorb Star® with the accumulated pyrolysis products were transported to the thermal desorption unit TDU2 from GERSTEL (Mülheim, Germany) using the MultiPurposeSampler (MPS) from GERSTEL (Mülheim, Germany). A gradient of 40°C/min from 40°C to 200°C was used for thermal desorption. The pyrolysis products were transported via helium stream through the transfer line (250°C) and cryofocused on a glass wool liner in the cooled injection system CIS4 from GERSTEL (Mülheim, Germany) at −100°C for 10 min. Subsequently, the pyrolysis products were released into the gas chromatographic system by heating the CIS unit from −100°C up to 270°C with a heating rate of 12°C/s in solvent vent mode with 30 mL/min purge flow to split vent at 0.02 min (comparable with 30:1 split injection).

#### 2.3.3 Gas Chromatographic Separation and Detection of Pyrolysis Products

To separate the analytes, we used an Agilent 8890 GC system (Santa Clara, USA) with an Agilent HP 5MS UI GC column (Santa Clara, USA). The pyrolysis products were separated from 40°C to 250°C at a heating rate of 3.5°C/min. To minimise the carry‐over effect, the oven was heated to 300°C at a rate of 50°C/min and held at 300°C for 5 min at the end of each GC‐run. The pyrolysis products were detected using an Agilent 5977 GC/MSD (Santa Clara, USA) single quadrupole mass spectrometer. The transfer line was set to 300°C, the ion source to 230°C and the quadrupole to 150°C. The mass spectra were obtained by measuring in full scan mode from 35 to 550 *m/z* after electron ionisation at 70 eV.

### 2.4 Data Analysis

The pyrolysis products were identified with NIST17 database and evaluated with different literature [19, 20, 21, 22]. An inhouse database includes pyrolysis products (PE, PET, PLA, PP, PS, PVC, SBR), impurities, plasticisers and matrix compounds. For data acquisition, qualitative and quantitative analysis MassHunter Workstation (Agilent, V 10.0) was used.

### 2.5 Method Development

To analyse the potential impact of matrix effects and adapt the methodology to plant material, three calibration curves were created with the same PS and IS concentrations (0.1–6 μg PS absolute, 8 calibration points) using different amounts of dried and ground plastic‐free pak choi powder (0, 5, 10 mg). The results of the matrix effect analysis indicated that 10 mg of plastic‐free dried pak choi powder should be mixed with PS absolute (0.1, 0.2, 0.3, 0.5, 0.7, 1.0, and 1.5 μg) to create calibration curves for the measurements. The analytical limits were obtained with a regression analysis. The LOD and LOQ were obtained by the standard deviation of the response and the slope of the calibration curve [23]. The repeatability of the method was evaluated by measuring eight samples within a day, each containing 3 μg of IS, 0.5 μg of PS and 10 mg of plastic‐free pak choi powder. The size‐dependent effect on PS pyrolysis was analysed by measuring the equal mass amount (5 μg of PS) of different‐sized PS MNPs (100, 500, 2000, and 5000 nm) in triplicate. To ensure complete thermal desorption, the temperature of the TDU was set to 240°C. For a better comparison of the results, 3μg ^13^C_6_‐PS was added to the samples as an IS.

## 3 Results and Discussion

### 3.1 Qualitative Analysis of PS

The main focus in this work is the qualitative and quantitative analyses of PS in plant material. After pyrolysis, thermal extraction, thermal desorption and GC separation of the PS reference material, the three main analytes (styrene monomer, dimer: 1,3‐diphenyl‐3‐butene, and trimer: 1,3,5‐triphenyl‐5‐hexene) according to the literature could be identified (Figure S2) [19, 20, 24]. Due to the release of styrene monomer from other plastics (SBR) or plant material, it is not a suitable marker for identification and quantification of PS [22]. For other common polymers (PE, PET, and PP), matrix spiked samples were analysed with this method and typical markers were identified (Table 1 and Figure S3). The presence of PS marker (trimer) interference could not be observed in other plant matrices (*S. lycopersicum, N. tabacum, S. europaea* and *A. officinalis* ), which makes this method also suitable for the analysis of other plants (see Figure S4).

**TABLE 1:** Markers used for polymer analysis (underlined ions used for quantitative analysis).

Polymer	Pyrolysis product	Abbreviation	Retention time (min)	Ions (m/z)
Polystyrene (PS)	Styrene (monomer)	PS1	6.3	51, 77, 78, 103, 104
	1,3‐Diphenyl‐3‐butene (dimer)	PS2	35.1	91, 104, 117, 130, 208
	1,3,5‐triphenyl‐5‐hexene (trimer)	PS3	54.0	91, 117, 194, 207, 312
Labelled ^13^C_6_‐PS (IS)	^13^C_6_^−^Styrene (monomer)	IS1	6.3	55, 83, 84, 109, 110
	^13^C_12_‐1,3‐Diphenyl‐3‐butene (dimer)	IS2	35.1	97, 110, 122, 136, 220
	^13^C_18_‐1,3,5‐Triphenyl‐5‐hexene (trimer)	IS3	54.0	97, 122, 206, 219, 330
Polyethylene (PE)	1,14‐Tetradecadiene	PE1	24.0	55, 81, 96, 109, 207
	1‐Nonadecene	PE2	40.2	83, 97, 111, 238, 266
	1‐Eicoscene	PE3	43.0	83, 97, 111, 252, 280
Polyethylene terephthalate (PET)	Divinyl terephthalate	PET1	30.2	104, 132, 147, 175, 218
Polypropylene (PP)	2,4,6,8‐Tetramethyl‐10‐undecene (Isomer 1)	PP1	21.1	69, 85, 111, 125, 210
	2,4,6,8‐Tetramethyl‐10‐undecene (Isomer 2)	PP2	21.4	69, 85, 111, 125, 210
	2,4,6,8‐Tetramethyl‐10‐undecene (Isomer 3)	PP3	21.7	69, 85, 111, 125, 210

Other polymers can be implemented in the existing method in future research, but were not in the focus of this study. Additionally, there are only a limited number of isotopically labelled reference materials of polymers, which limits the development, optimization and validation of methods for sampling, sample preparation and detection for other polymers. Despite this limitation, a first step towards the development of a methodology for the detection of MNPs in plant matrices has been made.

### 3.2 Quantitative Analysis of PS

In order to conduct a quantitative analysis, ^13^C_6_‐labelled PS was employed as the IS, given that the aforementioned matrix‐effected deuterium exchange reactions on the D_5_‐PS were observed [25]. The formation of the monomer is influenced by the quantity of matrix present, as evidenced by the low *R* ^2^ value of 0.462 for the calibration function (Figure S5). The dimer and trimer have proved to be suitable, whereas the highest *R* ^2^ value, which is independent of the amount of matrix, could be obtained with the trimer (*R* ^2^ = 0.994) and has been used for quantification purposes. A matrix calibration curve (10 mg of spiked MNPs free plant material) was generated using the styrene trimer (PS: quantifier: *m/z* 91, qualifier: *m/z* 312, *m/z* 207, IS: quantifier: *m/z* 97, qualifier: *m/z* 330, *m/z* 219). Following the initial sampling and subsequent calibration, the upper limit of the calibration range was reduced from 6 to 1.5 μg of PS. The linear working range is from 0.1 to 1.5 μg of PS absolute and was obtained by spiking 10 mg of plastic‐free dried pak choi powder with seven calibration concentrations (*R* ^2^ = 0.996). The calculated LOD was 0.09 μg of PS, and the LOQ was 0.28 μg of PS absolute in 10 mg matrix [23]. The obtained results were comparable with literature for TED‐GC/MS methods used for MNPs determination in soil [22, 26]. In comparison to pyrolysis GC/MS methods, our limits were about four times higher, but the sample preparation is much less time‐consuming and labour‐intensive. This difference can be attributed to the sample preparation, purification and concentration of the polymers described in the literature [9, 10].

**FIGURE 2:**
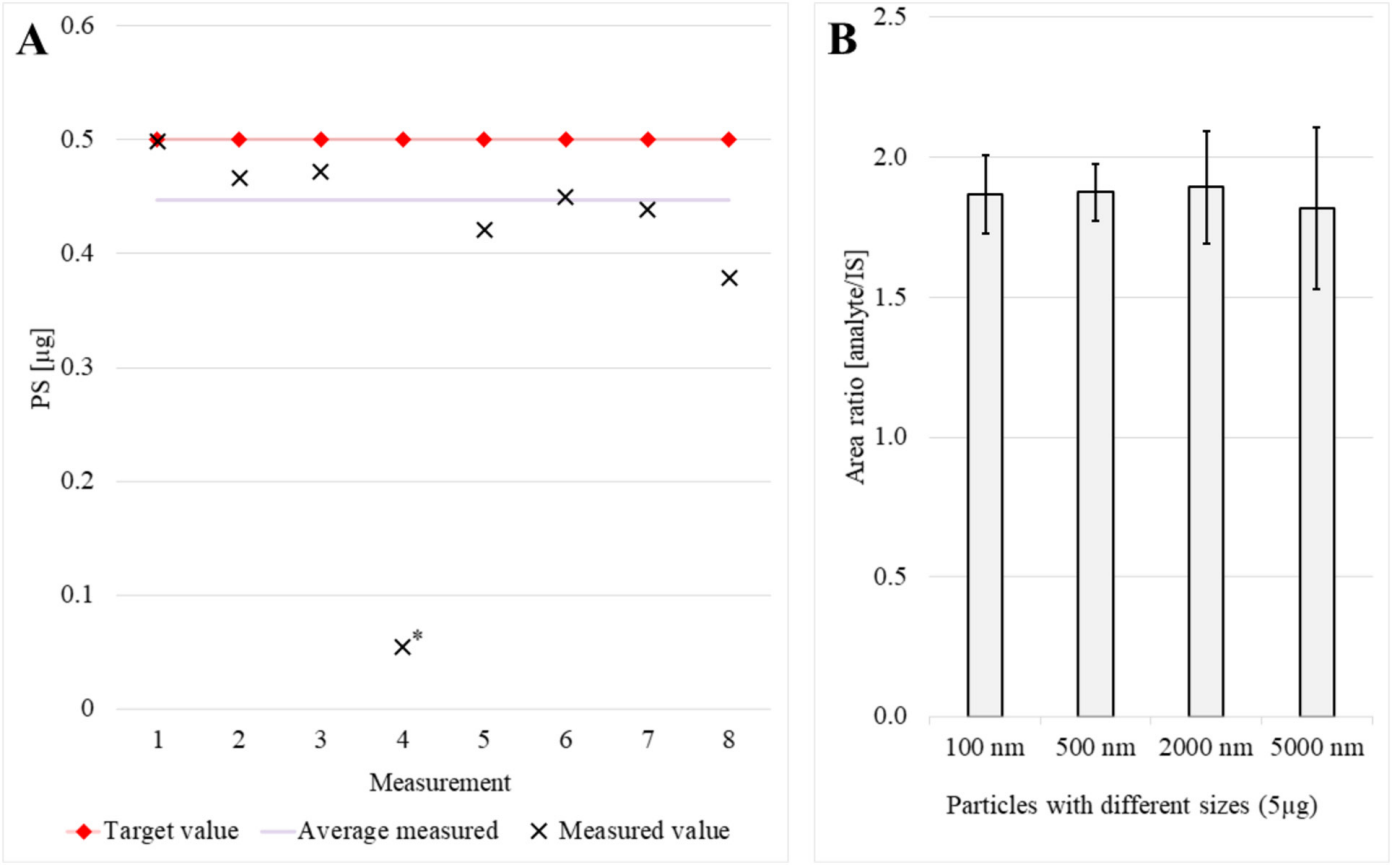
(A) Repeatability of the detection determined by the analysis of spiked (0.5 μg of PS) matrix samples (10mg). * is used to indicate an outlier (Grubbs test, *p* < 0.05). (B) Impact of particle sizes on the quantification result (100, 500, 2000, and 5000 nm).

The repeatability of the method was analysed by measuring eight spiked matrix samples (Figure 2B). One outlier was identified through the application of the Grubbs test (*p* < 0.05). This was determined to be an experimental error and was subsequently excluded from the calculation of the relative standard deviation (RSD) and systematic error (bias). The bias was determined to be −10.7%, with an RSD of 8.7%. These values are consistent with those reported in the existing literature [27].

### 3.3 Particle Size Effects

This is the first study, where the impact of the particle size on quantification MNPs was analysed. The analysis of the area ratios (MNPs/IS) revealed no significant differences (Figure 2B). This suggests that the particle size has no impact on the accuracy of our quantification method. In another study, the recovery of nanoparticles of different sizes (30, 200, and 700 nm) in water analyses was examined, and the findings are consistent with the results of our study [28]. Due to the size limitations of spectroscopic methods, the TED‐GC/MS method can be used for the detection and identification of MNPs [7, 29].

### 3.4 Further Method Optimisation to Reduce Carry‐Over

To reduce the carry‐over effect of the high boiling pyrolysis products such as the PS trimer, the transfer line from the TGA to the TAU and the other parts of the TAU were heated up to 250°C after each measurement (max. temperature for the technical equipment). The PS trimer evaporates from the inner surfaces and get transported with the gas stream from the TGA to the waste, which leads to a significant reduction of the carry‐over effect (Figure S6). As far as the authors are aware, there are no other publications in which the thermal cleaning of the transfer line after each measurement was investigated.

### 3.5 PS Content in Pak Choi

**FIGURE 3:**
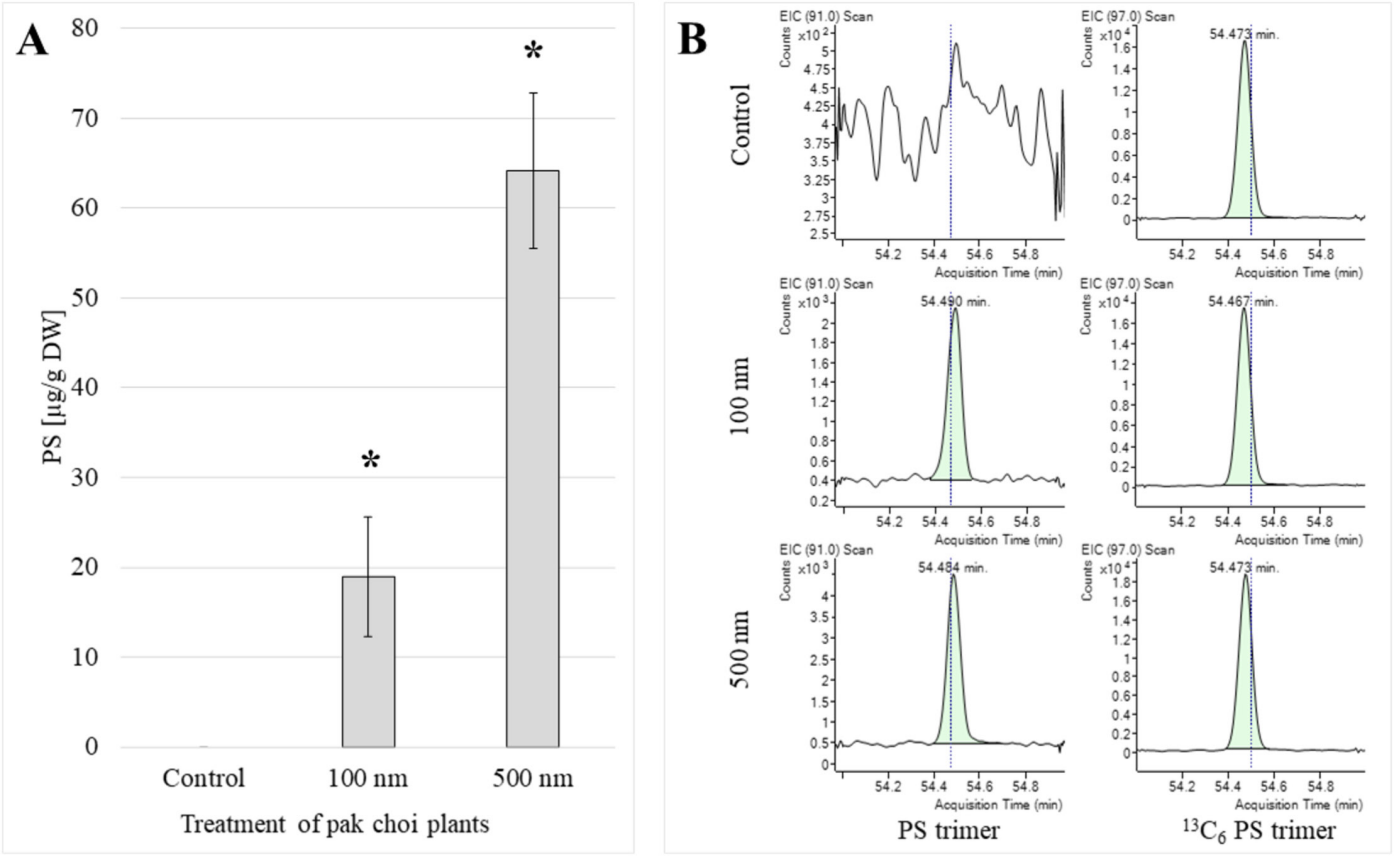
Results of the determination of PS in pak choi grown in 0.5 wt.% PS‐soil mix (control without PS, 100 and 500 nm); (A) quantitative analysis of PS in pak choi (mean ± SE, * significant difference vs. control (Dunn‘s method, *p* < 0.05); (B) Example of mass signal integration (left: analyte PS, right: IS).

The samples of pak choi grown in a PS‐soil mixture containing 0.5 *wt*.% PS (100 and 500 nm) were analysed using the developed method for analysing PS in pak choi. PS could be detected and quantified in the treated samples, while no PS could be detected in the control. The automatic integration of the EIC signals at RT 54.5 is illustrated in Figure 3B. On the left side, *m/z* 91 corresponds to the PS marker (trimer), while on the right side, *m/z* 97 corresponds to the IS (^13^C_6_‐PS marker, trimer). In the plants treated with 100 nm of particles, 19.0 ± 6.7 μg PS in 1 g of dried plant material and 64.1 ± 8.6 μg/g PS were found in the plants treated with 500 nm of particles. The mass concentration of PS in pak choi grown in a soil mixture containing 100 nm of MNPs is observed to be lower than that observed in pak choi grown in a soil mixture containing 500 nm of MNPs (Figure 3A). In comparison to PS contents found in other vegetables, our measured content was more than 10 times lower. Up to 1083 μg/g PS was found in cucumber leaves using a pyrolysis GC/MS method. Due to the different sample preparation, experimental setup (grown in soil vs. hydroponic conditions) and plant species, the data can hardly be compared [9]. In another study, no PS could be detected in the crop plants, but other polymers could be found in cowpea (485.1 μg/g sum average concentration of PE, PVC, PET and polyamide‐66) and in flowering cabbage (264.8 μg/g) analysed with a pyrolysis GC/MS method [10]. The utilisation of an external calibration curve in conjunction with pure reference polymers has the potential to result in an overestimation or underestimation, provided that the process of sample extraction does not ensure the complete removal of all organic matrix residues [9, 10]. The matrix effects for the TED‐GC/MS method were reduced due to the matrix spiked calibration curve and the use of an ^13^C_6_‐PS IS [26]. The great differences in the results of the studies using different analytical methods emphasise the need for harmonisation of analytical methods for a better comparison of the data and the assessment of risk due to human consumption.

Given the known dimensions of the PS particles (100 and 500 nm) in our model study, it is feasible to estimate the quantity of particles that have been taken up by the plant (Figure S7). In contrast to the determination of the mass content, the calculated particle amount suggests that particles with a diameter of 100 nm were taken up in greater quantities than particles with a diameter of 500 nm. It is essential that information regarding the particle composition is made available or detected by a complementary method for the purpose of facilitating a meaningful comparison between the measurement data.

### 3.6 Analytical Limitations

Thermoanalytical methods are destructive techniques [7]. This method is not suitable for analysing the shape and size of the MNPs or the location of the particles in plant tissues. In order to address these limitations, complementary methods such as CLSM, SEM or other spectroscopic methods are required [8, 11]. However, these methods are not appropriate for the analysis of nanoparticles, as the resolution limits of spectroscopic methods (μ‐Raman: particle size > 1 μm, μ‐FTIR: particle size > 10 μm) are inadequate. In microscopic methods, particles are doped with fluorescent dye or metal ions so that they can be detected, but this is not useful for field samples [7, 30]. The MNPs are susceptible to alteration as a consequence of the natural ageing processes that occur in the environment [31]. The impact of qualitative and quantitative analysis of aged MNPs has yet to be investigated using this method. PTFE was excluded from analysis, because the pyrolysis product tetrafluoroethylene, mentioned in the literature, could not be detected [19]. PE is another polymer which is hardly detectable in plant matrix. Due to the chemical similarity of thermal decarboxylated fatty acids to PE, it is not feasible to ascertain the source of the typical alkene and alkane marker signals (Figure S8). Dialkenes (e.g., 1,14‐Tetradeca‐diene) are specific markers for the thermal degradation of long chained hydrocarbons like in PE. Nevertheless, these are only formed in a minimal quantity, which leads to a high LOD [32, 33]. The marker lactide (3,6‐dimethyl‐1,4‐dioxane‐2,5‐dione) is mostly used for the detection and quantification of PLA [10, 21, 26]. However, it should be noted that lactide is also formed in the presence of the monomer lactic acid in the matrix (Figure S9).

## 4 Conclusion

In this study, a new TED‐GC/MS method was developed for the detection and quantification of PS in pak choi. In addition to the qualitative and quantitative analysis, it was possible to gain further insights into the influence of the particle size of MNPs. It is the first time that this analytical approach has been used for the analysis of MNPs in a plant matrix and provides an important basis for risk assessment of MNPs in vegetables. However, the findings also underscore the urgent necessity for the development of harmonised and validated methodologies.

## Author Contributions

**Zytowski E:** writing – original draft, investigation, conceptualization, project administration, methodology. **Baldermann S:** writing – review and editing, supervision, project administration, funding acquisition.

## Conflicts of Interest

The authors declare no conflicts of interest.

## Supporting information

Figure S1: TGA curves of different polymers (polytetrafluoroethylene (PTFE), polyethylene (PE), polypropylene (PP), polyethylene terephthalate (PET), polystyrene (PS), polyvinylchloride (PVC), styrene butadiene rubber (SBR)) for method optimization. The blue area indicates the sampling range for thermal extraction.
Figure S2: The figure represents the mass spectra including the fragments of the three main markers in pyrolysis of PS.
Figure S3: Polymer spiked matrix samples (plastic‐free, dried pak choi powder).
Figure S4: Different matrix samples tested for PS marker (PS3: 1,3,5‐triphenyl‐5‐hexene). A: Complete TIC chromatograms of different plant samples. B: Zoomed in at RT 54.5 min for checking the PS3 marker.
Figure S5: Calibration curves of the PS marker (monomer: styrene, dimer: 1,3‐diphenyl‐3‐butene, and trimer: 1,3,5‐triphenyl‐5‐hexene) depending on the mass of spiked, plastic‐free, dried pak choi powder.
Figure S6: Reduction of carry‐over effect of ^13^C_6_‐PS trimer by heating out thermal absorption unit (TAU). 2 different, comparable experiments were measured with different post measurements conditions. A: TAU was raised by 10° from 240°C to 250°C for 10 min after each run. B: TAU was kept under same conditions (240°C).
Figure S7: Number of particles in 1 g dried plant material. A: Calculated amount of uptaken particles depending on different treatments (pak choi grown on soil 100 nm of PS MNPs or 500 nm of PS MNPs). B: Calculation of particle amount based on mass‐based data shown in Figure 4.
Figure S8: Chromatograms with markers for the pyrolysis products of various saturated fatty acids (C_15_H_31_COOH, C_17_H_35_COOH, and C_19_H_39_COOH) and PE.
Figure S9: The mass spectra at the retention times of 15.40 min (PLA) and 15.25 min (lactic acid) of 3,6‐dimethyl‐1,4‐dioxane‐2,5‐dione.

## Data Availability

The data that support the findings of this study are available from the corresponding author upon reasonable request.
