# Supplementary material for: Thermal Desorption and Extraction Coupled With Gas Chromatography and Mass Spectrometry for the Quantification of Polystyrene Nanoplastic in Pak Choi
Source: Rapid Commun Mass Spectrom. 2025 Apr 14;39(14):e10046. doi: 10.1002/rcm.10046 (PMC11994979; doi:10.1002/rcm.10046)
Supplement: Supplementary file 1 — Figure S1: TGA curves of different polymers (polytetrafluoroethylene (PTFE), polyethylene (PE), polypropylene (PP), polyethylene terephthalate (PET), polystyrene (PS), polyvinylchloride (PVC), styrene butadiene rubber (SBR)) for method optimization. The blue area indicates the sampling range for thermal extraction. Figure S2: The figure represents the mass spectra including the fragments of the three main markers in pyrolysis of PS. Figure S3: Polymer spiked matrix samples (plastic‐free, dried pak choi powder). Figure S4: Different matrix samples tested for PS marker (PS3: 1,3,5‐triphenyl‐5‐hexene). A: Complete TIC chromatograms of different plant samples. B: Zoomed in at RT 54.5 min for checking the PS3 marker. Figure S5: Calibration curves of the PS marker (monomer: styrene, dimer: 1,3‐diphenyl‐3‐butene, and trimer: 1,3,5‐triphenyl‐5‐hexene) depending on the mass of spiked, plastic‐free, dried pak choi powder. Figure S6: Reduction of carry‐over effect of 13C6‐PS trimer by heating out thermal absorption unit (TAU). 2 different, comparable experiments were measured with different post measurements conditions. A: TAU was raised by 10° from 240°C to 250°C for 10 min after each run. B: TAU was kept under same conditions (240°C). Figure S7: Number of particles in 1 g dried plant material. A: Calculated amount of uptaken particles depending on different treatments (pak choi grown on soil 100 nm of PS MNPs or 500 nm of PS MNPs). B: Calculation of particle amount based on mass‐based data shown in Figure 4. Figure S8: Chromatograms with markers for the pyrolysis products of various saturated fatty acids (C15H31COOH, C17H35COOH, and C19H39COOH) and PE. Figure S9: The mass spectra at the retention times of 15.40 min (PLA) and 15.25 min (lactic acid) of 3,6‐dimethyl‐1,4‐dioxane‐2,5‐dione. [file RCM-39-e10046-s001.docx]

**Supplemental material for**

**“Thermal desorption and extraction coupled with gas chromatography and mass spectrometry for the quantification of polystyrene nanoplastic in pak choi”**

Zytowski E and Baldermann S


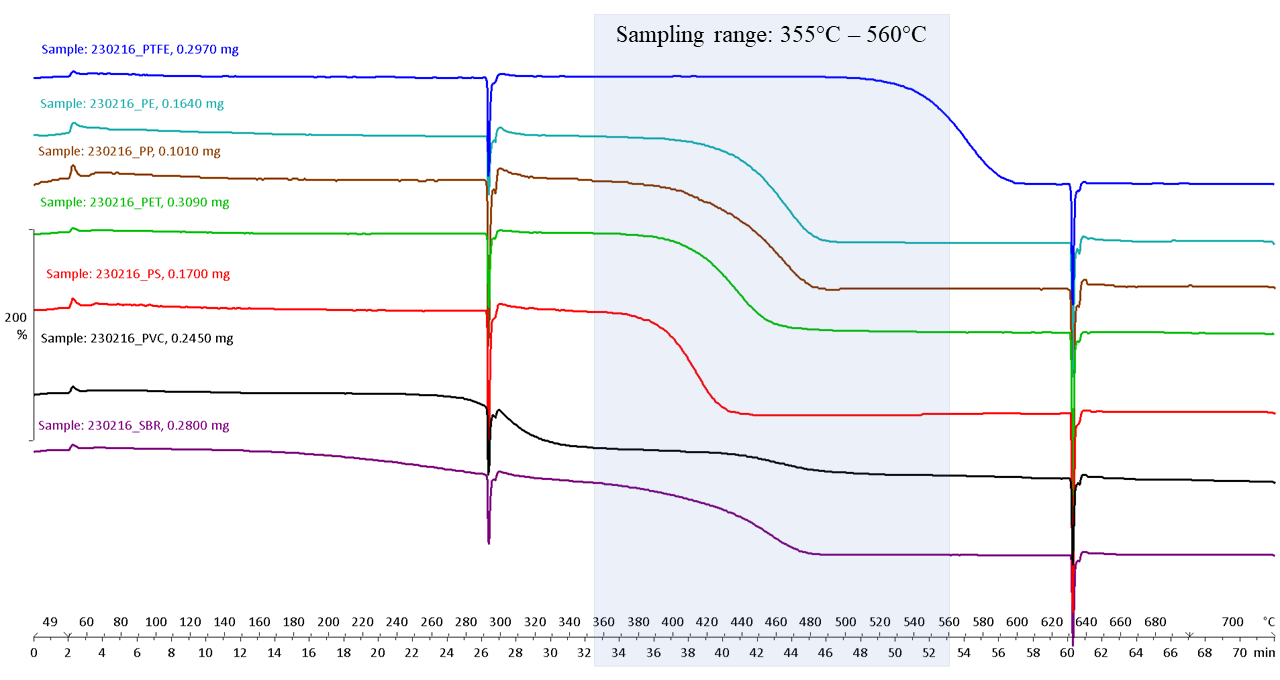


Supplemental Figure S1: TGA curves of different polymers (polytetrafluoroethylene (PTFE), polyethylene (PE), polypropylene (PP), polyethylene terephthalate (PET), polystyrene (PS), polyvinylchloride (PVC), styrene butadiene rubber (SBR)) for method optimization. The blue area indicates the sampling range for thermal extraction.


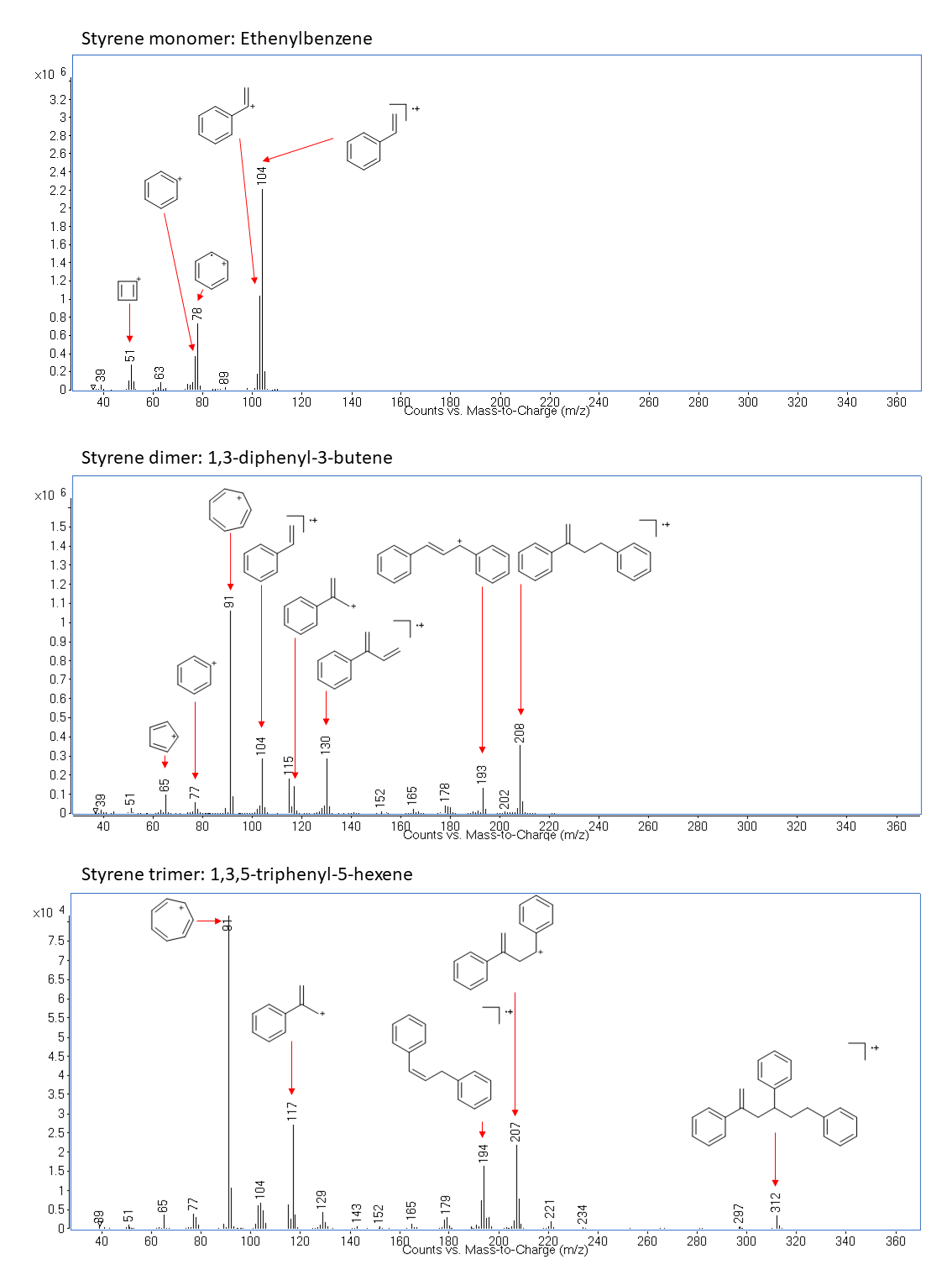


Supplemental Figure S2: The figure represents the mass spectra including the fragments of the three main markers in pyrolysis of PS.


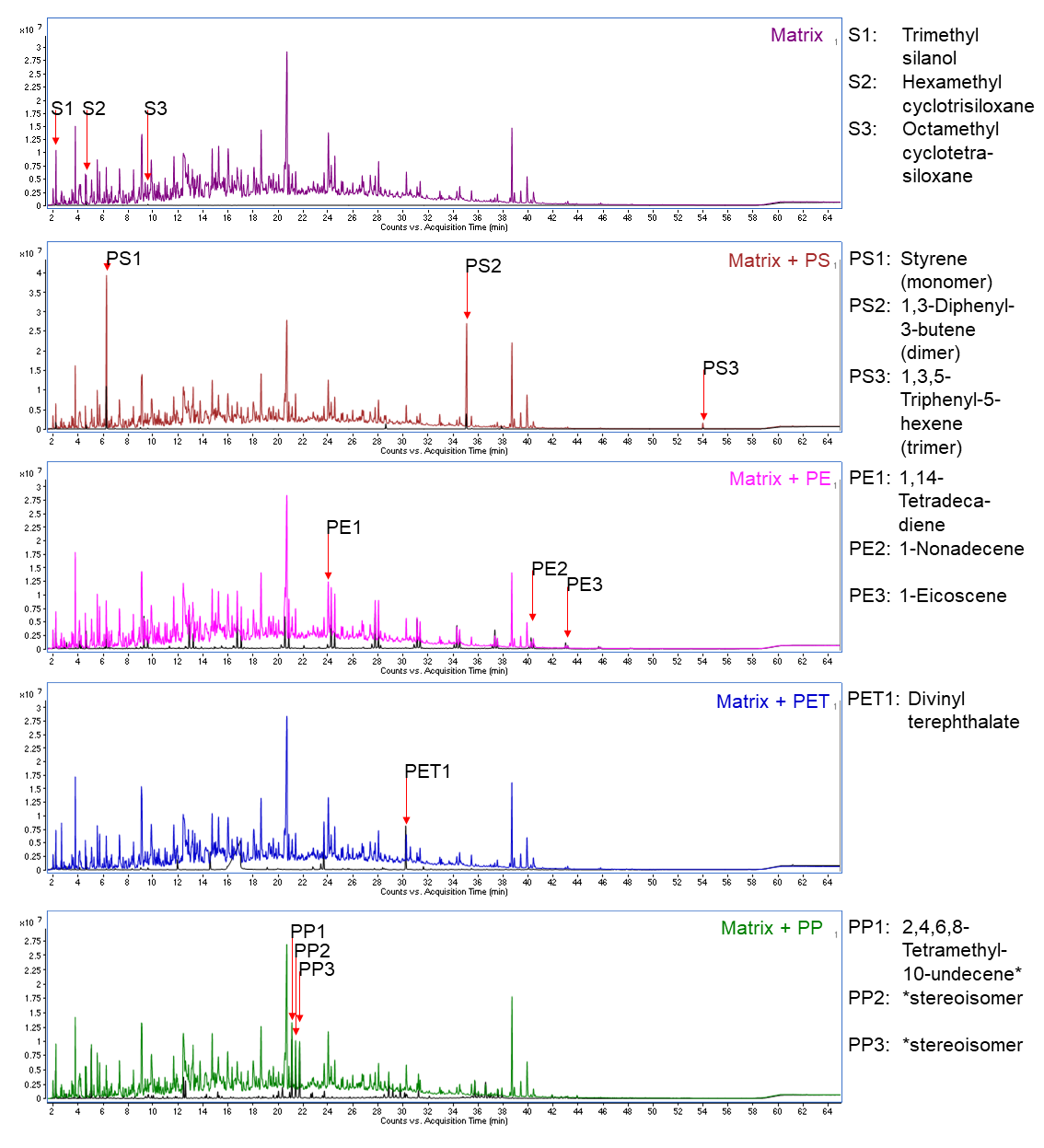


Supplemental Figure S3: Polymer spiked matrix samples (plastic-free, dried pak choi powder).


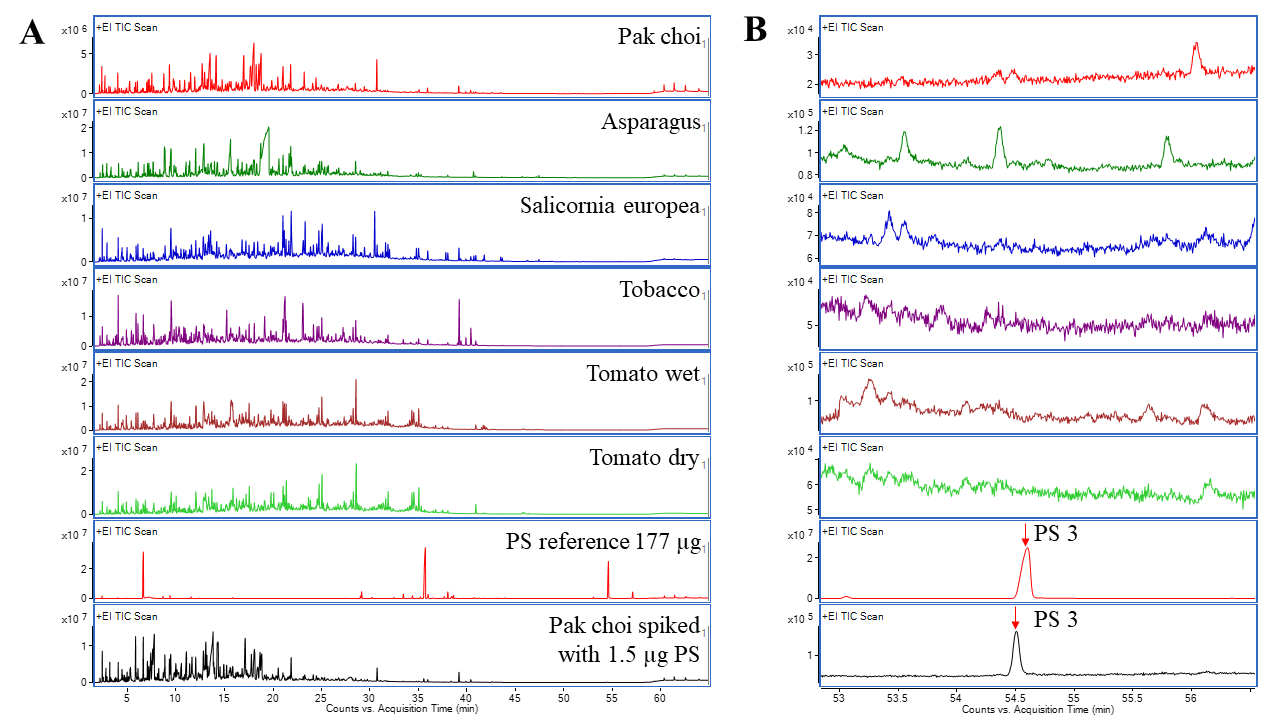


Supplemental figure S4: Different matrix samples tested for PS marker (PS3: 1,3,5-triphenyl-5-hexene). A: Complete TIC chromatograms of different plant samples. B: Zoomed in at RT 54.5 min for checking the PS3 marker.


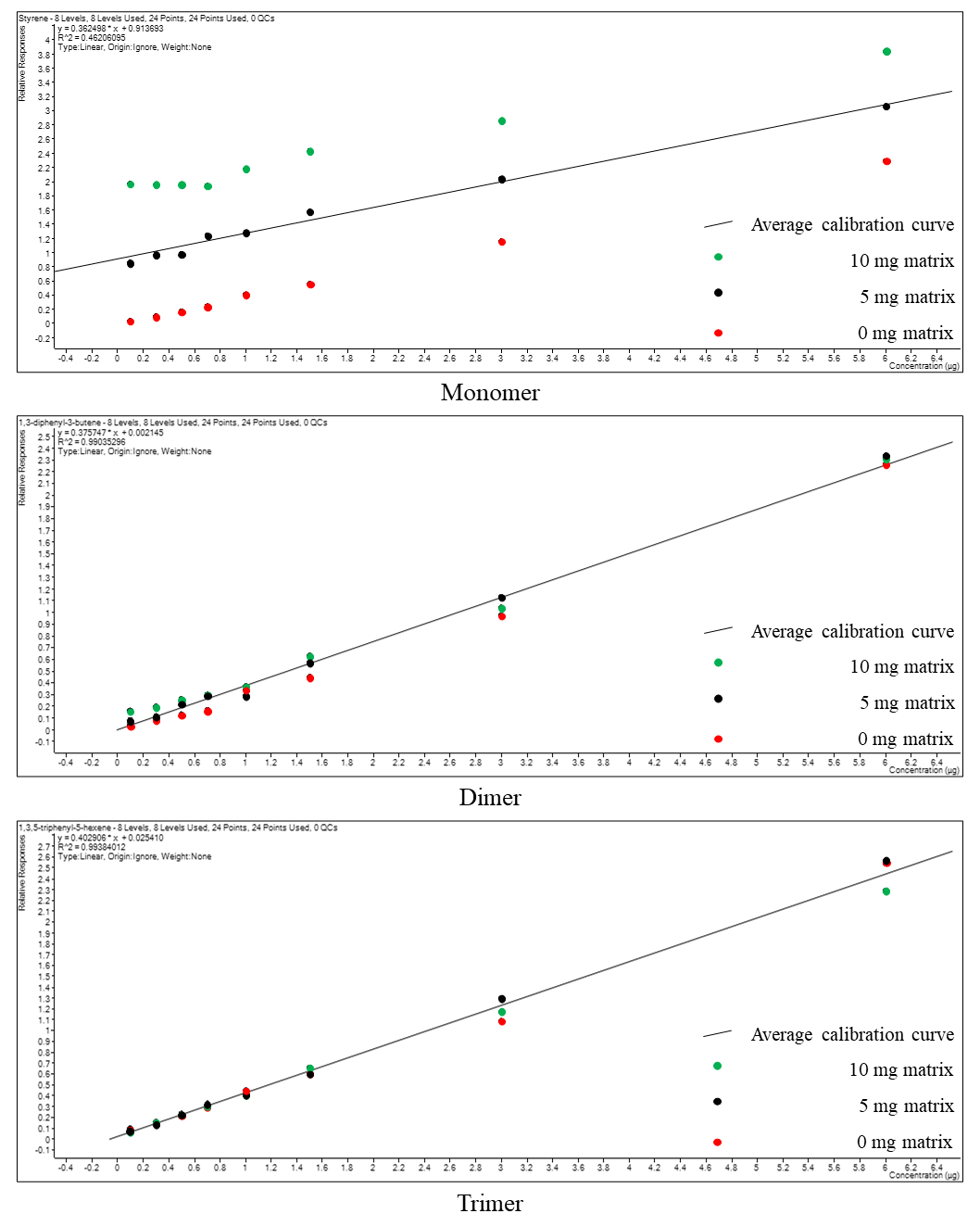


Supplemental Figure S5: Calibration curves of the PS marker (monomer: styrene, dimer: 1,3-diphenyl-3-butene, and trimer: 1,3,5-triphenyl-5-hexene) depending on the mass of spiked, plastic-free, dried pak choi powder.


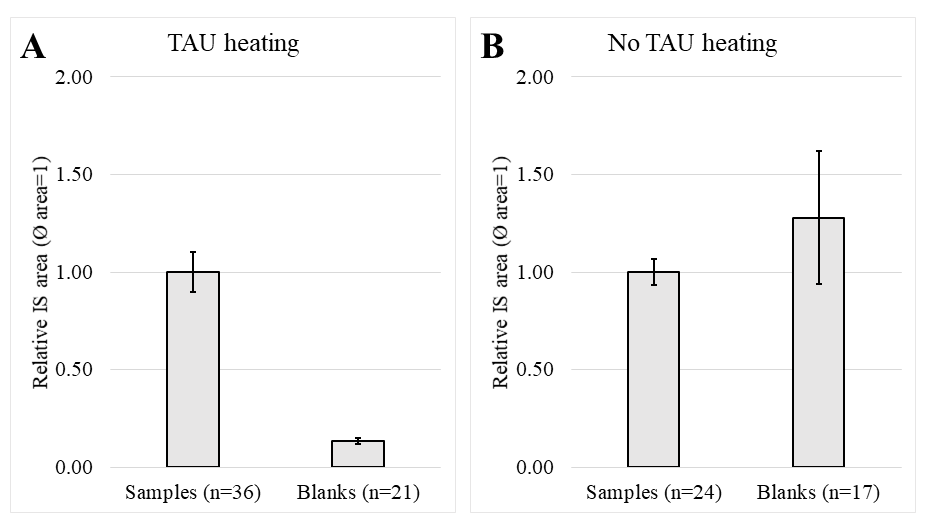


Supplemental Figure S6:Reduction of carry over effect of ^13^C_6_-PS trimer by heating out thermal absorption unit (TAU). 2 different, comparable experiments were measured with different post measurements conditions. A: TAU was raised by 10 degrees from 240°C to 250°C for 10 minutes after each run. B: TAU was kept under same conditions (240°C).


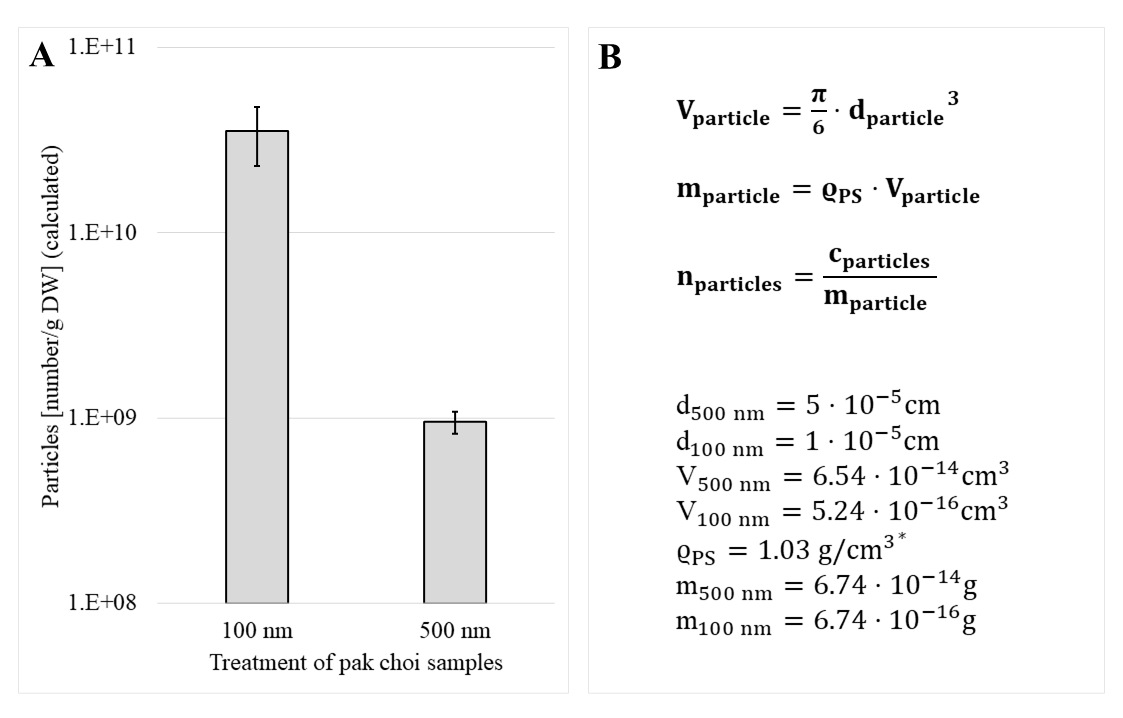


Supplemental Figure S7: Number of particles in 1 g dried plant material. A: Calculated amount of uptaken particles depending on different treatments (pak choi grown on soil- 100 nm PS MNPs or 500 nm PS MNPs). B: Calculation of particle amount based on mass-based data shown in figure 4.


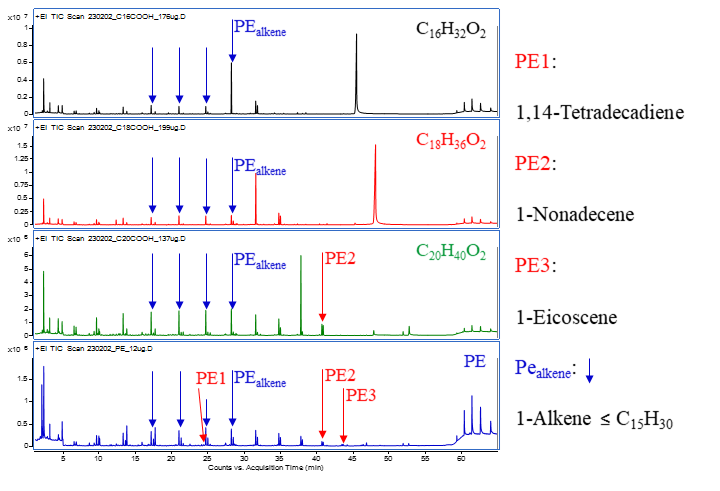


Supplemental Figure S8: Chromatograms with markers for the pyrolysis products of various saturated fatty acids (C_15_H_31_COOH, C_17_H_35_COOH, C_19_H_39_COOH) and PE.


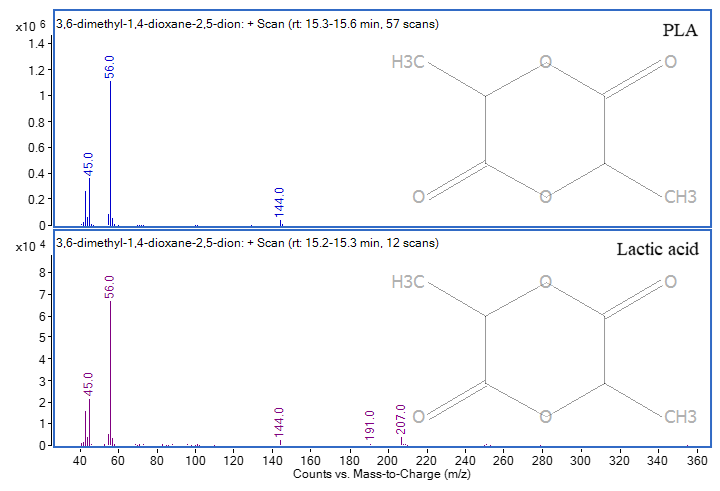


Supplemental Figure S9: The mass spectra at the retention times of 15.40 minutes (PLA) and 15.25 minutes (lactic acid) of 3,6-dimethyl-1,4-dioxane-2,5-dione.
